# “It’s Time to see What I Can Do”: A Mixed-Methods Investigation into Trajectories of Resilience in Adolescents during the COVID-19 Pandemic

**DOI:** 10.1007/s40653-024-00642-5

**Published:** 2024-06-05

**Authors:** K Fradley, K. M. Bennett, R. E. Ellis, J. Gibson-Miller, R. P. Bentall, L. Levita

**Affiliations:** 1 Department of Psychology, Edge Hill University, Lancashire, England; 2 Department of Psychology, The University of Sheffield, Sheffield, England; 3 Department of Psychology, University of Liverpool, Liverpool, England; 4 School of Education, The University of Sheffield, Sheffield, England; 5 School of Psychology, University of Sussex, Hove, England

**Keywords:** Resilience, Mental well-being, Adolescents, COVID-19 Pandemic, Mixed Methods

## Abstract

There is a concern that adolescent mental well-being and resilience has been adversely affected by the COVID-19 pandemic. Consequently, the aim of the current investigation was to track adolescents’ resilience from the initial months of the pandemic (T1) to approximately two years later (T2) using both qualitative and quantitative methods. Through interviews, thirty-one adolescents narrated their COVID-19 experiences and reflected on their mental well-being across this timespan. Using these accounts, we identified four groups of adolescents exhibiting one of the following trajectories of resilience: (1) Enduring resilience, (2) Reaching resilience, (3) Declining resilience and (4) Enduring non-resilience. Our findings revealed that most adolescents were able to maintain or develop good resilience on prolonged exposure to COVID-19 adversity (trajectories 1 and 2). This finding is contrary to the prevailing notion that the majority of adolescents’ mental well-being and hence resilience was adversely impacted by COVID-19 in the short and long term. Further qualitative analysis identified key factors that contributed to maintaining and developing greater levels of resilience during the pandemic: quality of friendships, quality of family relationships and regaining a sense of control. Lastly, we found a lack of congruence between quantitative and qualitative measures of mental well-being and resilience, suggesting that they might tap into different constructs/experiences. Significantly, our findings highlight that the majority of teens showed adaptive resilience during the pandemic and highlight the need for further longitudinal qualitative and quantitative research to assess both adaptive and maladaptive impacts of adversity on the adolescents’ mental well-being and resilience.

**Supplementary Information:**

The online version contains supplementary material available at 10.1007/s40653-024-00642-5.

## Introduction

The current study examined the presence and trajectory of resilience for adolescents’ mental well-being during the early and late period of the COVID-19 pandemic and aimed to identify key factors that encourage good mental well-being during times of adversity. Currently, some COVID-19 related research has given rise to the concern about the potential negative impact of the pandemic on the mental well-being of adolescents (Dewa et al., 2021; Imran et al., 2020; Lee, 2020; Orben et al., 2020; Widnall et al., 2022). Here we examine if this concern is justified, by exploring adolescents’ mental well-being and resilience at the start of the pandemic and two years later, in adolescents aged 13–16 at the start of the pandemic (April 2020).

Mental well-being is a broad and complex concept, operationalised as an individual’s everyday emotional, social, and behavioural functioning, and does not equate to solely the absence of a mental health disorder (Windle, 2009; World Health Organization, 2021). Poor mental well-being in adolescence predicts not only mental health disorders in later life (Fairchild et al., 2011; Paus et al., 2008), but also poorer educational attainment (Matthews et al., 2022; Wickersham et al., 2021) and employment outcomes (Hale et al., 2015). Adolescence, a prolonged period, spanning from puberty until the mid-twenties (Sawyer et al., 2018). During the COVID-19 pandemic adolescents faced disruptions in their education, social and family interactions and extracurricular activities. This disruption could be particularly impactful during this developmental period given that adolescence is associated with significant physical, hormonal neurobiological and psychological changes, in their transition to adulthood (Fairchild et al., 2011; Paus et al., 2008). It is also a key period for cultivating independence, nurturing friendships, and moulding self-identity (Albarello et al., 2018; Hay & Ashman, 2003; Roach, 2018; Spear & Kulbok, 2004; Steinberg & Morris, 2001). Consequently, it has been suggested that disturbances by the COVID-19 pandemic during this crucial developmental phase may contribute to future impairments in social, emotional, and behavioural functioning, potentially heightening vulnerability to psychopathology (Montero-Marin et al., 2023; Orben et al., 2020).

However, whether COVID-19 related disruptions during adolescence have impacted adolescents’ mental well-being and to what extent they were able to undergo a process of resilience (the ability to adapt to adversity) in the short and longer term is still a subject of debate. There is a need to understand the experiences of adolescents, as not all adolescents aged between 11 and 16 experienced poorer mental well-being as a consequence of COVID-19 in 2020 (82.40%; Vizard et al., 2020). Furthermore, preliminary findings from the C19PRC (Levita et al., 2020) found that younger age groups (aged 13–17) reported significantly lower depression and anxiety compared to older young people (aged 18–24) during the COVID-19 pandemic. As to why young people aged between 13 and 17 experienced less comparable mental health difficulties is currently unknown. To our knowledge, there has been no research to date that attempts to understand to what extent resilience occurred in adolescents aged between 13 and 17 during their exposure to pandemic-related adversity, and how it changed over time, from the early days to the later days of the COVID-19 pandemic. Therefore, the current study aimed to qualitatively identify resilience and understand how this changed amongst adolescents across two distinct time points during the COVID-19 pandemic: during the initial wave of the pandemic (April 2020; aged: 13–16) and two years later (January to May 2022; aged: 14–17).

There is no agreed operational definition for identifying adolescents as resilient or not from qualitative data. Bennett et al. (2022) were able to identify individuals in older populations (mean age of 51.46) as ‘resilient’ or ‘non-resilient’ at three timepoints across the COVID-19 pandemic, based on current theory and definitions of resilience (Windle, 2011). Here, resilience was broadly conceptualised as an adaptive process to manage adversity or stress using available internal and, or external resources. This aligns with the social ecological perspective, which proposes that young people actively navigate and negotiate available internal, and/or external resources to manage, counteract, or outweigh the effects of adversity or challenge (Bronfenbrenner, 1979; Ungar, 2011). We therefore used a similar criterion to identify resilience in adolescence and assumed that resilience was achieved when adolescents did not experience or report poor mental well-being; were able to maintain/return to a life that has meaning and/or satisfaction; and were actively participating in everyday life. Using this approach, we identified resilience in adolescence during the initial wave of the COVID-19 pandemic (time 1; T1, Age: 13–16), and approximately two years later (time 2; T2, aged: 14–17). Trajectories of resilience then emerged that allowed insights into those who had shown ‘enduring resilience’, ‘reached resilience’, ‘declining resilience’ and ‘enduring non-resilience’ across a two-year period (see Methods for details of classification).

Once identified, the current investigation highlighted factors that shaped resilience trajectories in adolescents during the pandemic. Whilst scarce, COVID-19 research today offers some support for the social ecological perspective in adolescents’ resilience. Many children and adolescents (aged 5–14) reported how they were active in reaching out for what they needed psychologically or socially from mutually available and trusting peers (Larivière-Bastien et al., 2022; Widnall et al., 2022). However, more research is needed to determine how adolescents were able to navigate and negotiate with friendships and other social resources during this difficult period. Although the current literature focuses on a wide age range (2–30 years), family support (Lips, 2021; Tso et al., 2020) and healthy coping mechanisms (Dewa et al., 2021) have also been shown to support good mental well-being during the COVID-19 pandemic but similarly require further study specifically in adolescence. Therefore, the present investigation aimed to build upon the currently limited literature by identifying which (if any) internal and, or external factors changed during the pandemic and contributed to resilience in adolescence ages between 13 and 16 at the start of the pandemic.

Finally, another key focus of this study was to investigate if qualitative (interviews) and quantitative (self-reports) methods of assessing adolescents’ mental well-being and resilience during the pandemic were concordant. Much of the existing studies on the impact of COVID-19 have used self-report quantitative measures for this purpose for children and young people aged between 2 and 24 (Creswell et al., 2021; Kauhanen et al., 2022). There have been no attempts to understand whether these measures accurately portray the experiences of adolescents, creating uncertainty about the validity of existing findings. It is important to know if such measures are able to assess adolescents’ mental well-being and resilience accurately, not least because UK policies have been informed by data collected through such measures (Office for Health Improvement and Disparities, 2022). Hence, a comparative analysis was undertaken to ascertain the alignment between self-reported data and qualitative assessments regarding the mental well-being and resilience of young individuals across various time points.

In summary, the current study utilised both qualitative and quantitative methods to gather data on the psychosocial impact of COVID-19 on a representative sample of adolescents aged 13–16 from an existing panel survey conducted one month into the first national lockdown (April 2020) and two years after the first survey (January to May 2022). Specifically, the present investigation aimed:

1. To gain a longitudinal qualitative understanding of the trajectories of resilience in adolescents between the initial (T1) and later phases of the COVID-19 pandemic (T2).
2. To identify key factors that encouraged good mental well-being in adolescents during the pandemic using qualitative data.
3. To examine the concordance between quantitative and qualitative measures of adolescents’ mental well-being and resilience.

## Methods

### Design and Recruitment

Qualitative and quantitative data were collected as part of the COVID-19 Psychological Research Consortium (C19PRC) Study (https://www.sheffield.ac.uk/psychology-consortium-covid19). The sample was taken from a panel survey on the impact of COVID-19 on young people aged 13–24 living in the UK, which was conducted one month in to the first national lockdown (henceforth known as Time 1, T1). Participants were recruited via the survey company Qualtrics (Qualtrics, 2020) between 21 and 29 April 2020, during a peak in positive COVID-19 cases and deaths (UK Government, 2021). In total, 2002 young people were recruited. When respondents endorsed a gender categorisation other than male or female, their responses (nonbinary, other and prefer not to say) were treated as missing data for the purposes of the current study, resulting in a final sample size of 1971. We contacted a sub-sample from this original cohort approximately 22 months later (January to May 2022; henceforth known as Time 2, T2). All the data collected is freely available through the UK data service (identification code: SN 9018). From the 643 eligible participants who had consented to be contacted for further research, a selected sub-sample aged 13–16 at T1 and therefore 18 or under at T2 were invited to participate in a semi-structured interview (*n* = 212) and a follow-up survey. Participants were asked to complete the online survey within a two-week time window after their interview. A sampling frame was used to ensure that the qualitative data was broadly representative of adolescents from different backgrounds: ethnic minorities and/or low socioeconomic status; to that end, a staggered approach to recontacting the participants was used. First, we contacted participants with protected characteristics (ethnic minorities and low SES; *n* = 82) and these individuals were invited first to complete a virtual semi-structured interview before we extended the invitation to adolescents from white and middle to high socioeconomic backgrounds (*n* = 136). SES status was classified using the socioeconomic index (for details, see http://fingertips.phe.org.uk/).

During recruitment for T2, several eligible participants did not wish to take part in a virtual person-led interview. To address this, we amended our protocol to also include an option to complete a written format of the interview. This survey consisted of open-ended items reflecting the questions asked in the verbal interviews, to give an option to adolescents to either have a person-led interview or respond online. In this study ten participants took part in a person led interview, and 22 completed the online version. For the interviews (verbal or written) and survey, all participants were compensated with £35 Love2Shop or Amazon vouchers.

#### Sample

For the current investigation, demographic data will only be presented for those who completed the follow-up interview at T2 (*n* = 31). Based on the quantitative data collected at T1, adolescents (who were interviewed) were aged 13–16 years (M = 15.69, SD = 1.12) during the initial months of the pandemic. The majority were female (58.06%, *n* = 18). The sample was predominantly White British/Irish at 83.87% (*n* = 26) (Black/African/Caribbean/Black British: *n* = 3, 9.68%; Asian/Asian British: *n* = 2, 6.45%). Based on the socioeconomic index (for details, see http://fingertips.phe.org.uk/), 29.03% (*n* = 9) of the sample were likely to be from a background of low SES, and the remining 70.97% (*n* = 21) were likely to be from a background of middle or high SES. Based on the quantitative data collected at T2, adolescents who were interviewed were aged between 14 and 17 (M = 15.68, SD = 1.11), approximately two years after the initial wave of the pandemic (January to May 2022). Gender reported at T2 remained the same for all adolescents who were interviewed.

### Ethical Considerations

Ethical approval was obtained from the University of Sheffield Ethics Committee (Number: 038532). All participants provided informed consent. Depending on the age of the participant (any individual < 16), a parent or guardian’s informed consent was also obtained prior to their participation. Ethics amendments were also approved for the written interview (open-ended survey).

### Measures

#### Verbal Semi-Structured Interviews

During the verbal semi-structured interview, participants were asked questions about their lives before the pandemic, during the pandemic, and what their life was like at the time of the interview. Finally, the adolescents were asked to reflect on the pandemic (Supplementary Information 1: Verbal interview guide). Grounded theory was adopted throughout data collection and analysis (Charmaz, 2006), and it is common practice to amend or include further questions based on the ideas that emerge during data collection; therefore, the interview guide changed throughout. For example, questions were later added to ask adolescents whether exposure to, or actively keeping up to date with the news impacted their mental well-being during the first few months of lockdown, and how this changed as lockdown persisted. All verbal interviews were either conducted online via google meet or telephone. Interviews approximately lasted between 45 min to an hour. For most of the interviews, to the researcher’s knowledge, no-one else was present, yet, for some interviews a family member of the young person, usually parent or guardian, was in the background and provided little to no input on the interview (*n* = 3). Many of the verbal semi-structured interviews were conducted by KF (*n* = 8), and the remaining was conducted by RE (*n* = 2). Both KF and RE were females and post-doctoral researchers at the time of the interviews; with RE moving onto a lecturer position during data collection. KF had prior qualitative experience by conducting focus groups and interviews with healthcare workers. Prior to recruitment, both RE and KF trailed the interview guide with a volunteer aged 17. Prior to the interview questions, the interviewer introduced themselves as well as stated the study’s aims and goals: to understand their experience of the COVID-19 pandemic and the impact it may have had on them. The verbal interviews were recorded (audio only) and transcribed via a third-party organisation (Devon Transcription).

#### Written Interview Online

The written interview was an open-ended survey which reflected the verbal interview (Supplementary Material: Written interview questions). Questions were broadened to ensure that exploration was maintained to allow adolescents the agency to talk about what was important to them. Unlike the verbal interviews, changes were not made to the written interview questions. Overall, due to the nature of the recruitment strategy, the interview process stopped once all eligible participants had been invited to take part, not once the researcher reached saturation.

#### Online Survey

The first survey (April 2020; T1) was delivered using Qualtrics (2020) and consisted of questionnaires and items designed to capture the experience of adolescents during the initial months of the pandemic. The follow-up T2 survey (January to May 2022) was delivered using a survey design created through Qualtrics (2020) and Gorilla (Anwyl-Irvine et al., 2020). The follow-up survey was amended to collect data suitable for the context at the time and included a Social Learning Test (Will et al., 2017) (for a full list of items within the T1 and T2 surveys see Supplementary Information 2). In this study, however, we report the data collected through the short-form Warwick-Edinburgh Mental Well-being Scale (SWEMWBS, Stewart-Brown & Janmohamed, 2008) and the Child and Youth Resilience Measure (CYRM; Ungar & Liebenberg, 2011) at T1 and T2.

The SWEMWBS (Stewart-Brown & Janmohamed, 2008) is a seven item scale on which participants were asked to rate the items on how best they described their experience over the previous two weeks. Items include ‘I’ve been feeling optimistic about the future’ and ‘I’ve been dealing with problems well’. Responses were on a five-point scale (1 = none of the time, 5 = all of the time) with higher scores indicating better mental well-being. Scores were summed and converted into a metric score using the table established for the SWEMWBS (Stewart-Brown & Janmohamed, 2008). In this sample internal reliability was high at both T1 and T2 (T1 alpha = 0.78; T2 alpha = 0.80), which support previous literature (alpha = 0.90, McKay & Andretta, 2017).

The CYRM (Ungar & Liebenberg, 2011) is a seventeen item scale. Items include ‘I get along with people around me’ and ‘I feel supported by my friends’. Responses were on a five-point scale (1 = not at all; 5 = quite a lot). Responses were summed and higher scores indicate greater resilience. In this sample internal reliability was high at both T1 and T2 (T1 alpha = 0.93; T2 alpha = 0.94).

### Data Analysis

#### Identifying Trajectories of Resilience in Adolescents across the COVID-19 Pandemic

Grounded theory (Charmaz, 2006) was adopted throughout data collection and analysis. Initial analysis was performed during data collection through post-interview memos and a research journal. After data collection, line-by-line coding was performed by KF using NVIVO (Released in March 2020, QSR International Pty Ltd., 2020). KF then generated focused codes, followed by axial codes. To improve the rigor of the analysis, a moderation meeting was performed to ensure that KF’s conceptual framework was in line with the other authors (RE and LL). Three transcripts were randomly selected and analysed by KF before the meeting. Authors (RE and LL) then discussed their interpretation of the narrative expressed in such transcripts and it was concluded that all authors agreed.

Using adolescents’ accounts of their mental well-being during the initial phase of the pandemic (T1) and two years after the initial phase of the pandemic (T2) during the time of the interview, adolescents were identified as reporting ‘resilience’ or ‘non-resilience’ independently at time 1 and time 2. The criteria has been adapted from similar studies that had identified individuals as ‘resilient’ or ‘non-resilient’ (Derrer-Merk et al., 2022; Donnellan et al., 2015) and therefore, adolescents were qualitatively identified as ‘resilient’ if they:

- Did not experience poor mental well-being.
- Were able to maintain/return to a life that has meaning and/or satisfaction.
- Were actively participating in everyday life.

Adolescents who did not meet this criterion were identified as ‘non-resilient’ for that time point. Categorisation at both T1 and T2 was performed by KF. Using the identification at T1 and T2, trajectories of resilience were categorised: ‘Enduring resilience’ (Resilient at T1 and T2); ‘Reaching resilience’ (non-resilient at T1 and resilient at T2), ‘Declining resilience (resilient at T1 and non-resilient at T2), and ‘Enduring non-resilient’ (non-resilient at T1 and T2). These groups were analysed further to understand the differences accounting for the trajectories of adolescents’ mental well-being during the COVID-19 pandemic.

#### Identifying Factors that Encourage Resilience in Adolescents during the COVID-19 Pandemic

Further qualitative analysis was undertaken to identify factors that encouraged resilience in adolescence during the COVID-19 pandemic. Group comparisons were performed to highlight potential factors that encouraged adolescents’ resilience throughout the COVID-19 pandemic. Where appropriate, group comparisons were performed using the ‘Coding Matrix Query’ available in NVIVO. Analysis was performed by KF.

#### Comparing Self-Report Measures with Adolescents’ Qualitative Experience

Quantitative analyses were performed to investigate whether self-report measures (SWEMWBS and CYRM) where congruent with the qualitative experience reported by adolescents during the COVID-19 pandemic. Descriptives statistics were performed for SWEMWBS and CYRM scores at T1 and T2 for each identification of resilience (‘resilient’ and ‘non-resilient). Independent t-tests were performed to determine if adolescents identified as ‘resilient’ or ‘non-resilient’ at T1, and separately at T2 had significantly different SWEMWBS and CYRM scores. Although both parametric and non-parametric t-tests were performed due to small sample sizes, there were no differences between the tests results. Therefore, parametric results will be presented.

Once trajectories of resilience in adolescents were identified further quantitative analysis was performed to determine if the self-reported data was in accordance with adolescents’ qualitative accounts. Descriptives statistics and paired t-tests were performed for SWEMWBS and CYRM scores at T1 and T2 for each group (nonparametric tests were also conducted but are not reported as the results were similar).

## Results

### Tracking Trajectories of Resilience during the Early and Late Phases of the COVID-19 Pandemic

Using the qualitative data of the adolescents reflecting on their experiences at T1, 15 adolescents were identified as ‘resilient’ and 16 as ‘non-resilient’ at T1. At T2, 26 were identified as ‘resilient’ and 5 as ‘non-resilient’. Table 1 shows the cross-tabulation of those identified as resilient and non-resilient at T1 and T2. Supplementary Information 1: Resilience category for each interview.

**Table 1:** Number of adolescents identified as resilient or non-resilient at T1 and T2

	T2	
	Non-resilient	Resilient
T1		
Non-resilient	3	13
Resilient	2	13

*Note* T1 = April 2020, T2 = January to May 2022

Using the information from Table 1, four groups were identified: (1) ‘Enduring resilience’ (Resilient at T1 and T2, *n* = 13); (2) ‘Reaching resilience’ (non-resilient at T1 and resilient at T2, *n* = 13), (3) ‘Declining resilience (resilient at T1 and non-resilient at T2, *n* = 2), and (4) ‘Enduring non-resilient’ (non-resilient at T1 and T2, *n* = 3). Table 2 shows the number of those in each group, as well as the demographics and descriptives of SWEMWBS (well-being) and CYRM (resilience) scores for each group at T1 and T2. Followed by qualitative descriptions of each of the four groups identified.

**Table 2:** Demographics, SWEMWBS (well-being) and CYRM (resilience) scores across the four groups of resilience

			Age		SES			Ethnicity	Gender		SWEMWBS	scores	CYRM	scores
	N (%)	Time 1 M (SD)	Time 2 M (SD)	Low (%)	Mid (%)	High (%)	Majority (%)	Minority (%)	Female (%)	Male (%)	Time 1 M (SD)	Time 2 M (SD)	Time 1 M (SD)	Time 2 M (SD)
Enduring	13	14	15.23	5	5	3	11	2	7	6	23.38	25.18	71.00	81.50
resilience	(42)	(1.41)	(1.17)	(38.46)	(38.46)	(23.08)	(84.62)	(15.38)	(53.85)	(46.15)	(3.74)	(6.36)	(11.14)	(4.95)
Reaching	13	14.62	16.31	1	6	6	10	3	4	9	21.06	20.86	70.31	71.80
resilience	(42)	(0.65)	(0.85)	(7.69)	(46.15)	(46.15)	(76.92)	(23.08)	(30.77)	(69.23)	(3.77)	(4.83)	(13.98)	(13.91)
Declining	2	14.50	14.50	1	I	0	2	0	1	1	19.35	22.51	67.50	73.50
resilience	(6)	(0.71)	(0.71)	(50)	(50)	(0)	(100)	(0)	(50)	(50)	(1.94)	(3.57)	(3.54)	(10.61)
Enduring	3	14	15.67	I	2	0	3	0	1	2	20.22	22.21	71.33	69.50
non-resilience	(9)	(1)	(0.58)	(33.33)	(66.66)	(0)	(100)	(0)	(33.33)	(66.66)	(3.16)	(5.25)	(12.66)	(21.92)

*Note* Time 1 refers to April 2020; Time 2 refers to between January and May 2022 SES Socioeconomic status

#### Enduring Resilience

Thirteen out of thirty-one adolescents interviewed were identified to showing resilience at both time points (42%). Adolescents in the ‘Enduring resilience’ group reported that they were able to manage the challenges of the pandemic during the initial phrase and two years later. During the initial phase, adolescents described how they were either able to manage the short-term difficulties of the initial phrase of the pandemic or felt that they were not impacted by them. Interviewee 1126 said that “At first it was like boring and I kind of just got used to everything” (Male, 14 at T2, Black/African/Caribbean/Black British, Low SES, T1) and interviewee 1349 said that “It didn’t really change my daily life as I didn’t go out by myself” (Female, 17 at T2, White, Low SES, T1).

Adolescents in the ‘Enduring resilience’ group reported good mental well-being two years on from the initial phrase of the pandemic. Additionally, adolescents in this group stated that the challenges lead to a sense of new meaning about their life. Interview 1539 said “…the pandemic I think it’s made me realise that I need to grow up a bit to handle as bad situation if it’s ever going to happen” (Interview 1539, Female, 14 at T2, White, Mid to High SES, T2). Interviewee 916 further corroborated this feeling as they said, “I think that made me realise a lot of stuff about my mental health” (Female, 14 at T2, White, Mid to high SES, T2). Therefore, their experience of the pandemic led to positive changes in their thoughts and feelings about themselves.

### Reaching Resilience

Thirteen out of thirty-one adolescents interviewed were identified as those who were ‘Reaching resilience’ by time 2 (42%). These adolescents reported short-term poor mental well-being due to the challenges associated with the COVID-19 pandemic during the initial phase of the pandemic (T1). Interviewee 1261 said “I was really scared. I didn’t know what was happening was going to happen” (Female, 16 at T2, Black/African/Caribbean/Black British, Low SES, T1). However, these adolescents reported improved mental well-being two years on from the initial phase of the pandemic. The majority of adolescents (*n* = 10) people in this group described how their improved mental well-being was due to changes experienced in response to the COVID-19 pandemic. These adolescents reported changes in their thoughts, feelings, and, or relationships, and therefore, stated that they were able to adapt and manage pandemic-related challenges and adversities since the pandemic started.

These days, I am doing better than I was before. I have learnt to focus and take care of myself better and to be less insecure of myself. I have amazing bonds with my friends and have even met someone great (Interview 362, Female, 17 at T2, Asian/Asian British, Mid to High SES, T2).

In this group, there was a small group of adolescents (*n* = 3) who reported that their improved mental well-being was attributed to the lifting of the restrictions that were occurring at the time of the interview. These adolescents stated that their mental well-being significantly improved as the COVID-19 restrictions lifted and the nation began returning to ‘normal’. They were “glad to be able to go out a bit again” (Interview 569, T2) and they were “…enjoying leading a normal life” (Interview 1353, T2). One adolescent stated that their experience of lockdown provided them with a new appreciation for their unrestricted life:

I think I’ve come to the realisation that meeting people and socialising is really key to keeping your mental health good. So now if I ever had the chance to go out and I weren’t restricted I definitely would… I think I would go for more opportunities because I feel like well at that point I didn’t have any, I couldn’t do anything (Interview 1523, Female, 17 at T2, White, Mid to high SES, T2).

### Declining Resilience

Two out of out of thirty-one adolescents interviewed were identified as having declining resilience across the pandemic. These adolescents reported that they were not affected by the challenges experienced by adolescents during the initial phase of the pandemic (T1) but became adversely impacted by the COVID-19 pandemic over-time (T2). Interviewee 781 said “I didn’t think much to start with but as time went on I became very scared” (Male, 17 at T2, White, Low SES, T2). These two young people reported that as the nation and government continued to respond to the COVID-19 virus (for example, social isolation, restrictions, school closure) feelings of anxiety and fear developed over the course of the pandemic. Feelings of anxiety and fear became much worse as restrictions lifted and schools re-opened for both adolescents at T2. These two adolescents said that they were extremely anxious about going out and socialising again. Interviewee 781 said “I felt unsafe nd [sic] felt anxious after not going anywhere for so long (Male, 14 at T2, White, Low SES, T2).

### Enduring Non-Resilience

Three out of thirty-one adolescents interviewed were identified as non-resilient across both time points. These adolescents described extreme difficulties in managing the short-term challenges of the COVID-19 pandemic, and as a result reported poor mental well-being. Interviewee 986 said “…I wouldn’t stop thinking about it and I go the point where I took a panic attack over it” (Female, 15 at T2, White, Mid to High SES, T1). These adolescents said that their mental well-being remained poor two years on from the initial phase of the pandemic and reported that the COVID-19 pandemic changed their everyday life. They also reported that their general mood was worse compared to before the COVID-19 pandemic and that everyday activities they used to enjoy did not feel the same. Interviewee 986 said “Well, before the pandemic I was happy, but now I’m dour” (Female, 15 at T2, White, Mid to High SES, T2). This was further corroborated by Interviewee 779 who said “We just had Easter and had a family day out but not as good as we used to have (Male, 16 at T2, White, Low SES, T2).

### Factors that Encouraged Resilience throughout the COVID-19 Pandemic

By understanding the similarities and differences across the groups, adolescents reported three key factors that encouraged resilience: quality of friendships; quality of family relationships; and regaining a sense of control.

#### Quality of Friendships

During the initial phase of the pandemic, adolescents who experience resilience at T1 and/or T2 (‘Endured resilience’, Reaching Resilience’ and ‘Declining resilience’) reported how they attempted to maintain their friendships during the COVID-19 pandemic through digital means (facetime, messaging, social media). However, across all the groups there were clear barriers for adolescents in maintaining their friendships as lockdown persisted. Adolescents reported that technology was an ineffective alternative to face-to-face communication and interaction for maintaining friendships across the pandemic. Interviewee 1261 said “I was still texting them and still FaceTiming them and stuff, but I just wanted to see them and just go out how we used to” (Female, 16 at T2, Black/African/Caribbean/Black British, Low SES, T1).

There was a preference to interact with their friends face-to-face, especially during the later months of the pandemic. All adolescents felt that the lack of peer interaction increased feelings of boredom and for some, loneliness, especially as lockdown persisted. In one case, the desire or need to see friends face-to-face was strong enough to break lockdown rules at least once (Interview 454). Additionally, the lack of peer interaction was reported to have a long-term impact on adolescents’ pre-pandemic friendships and led to a reduction in their social circle during and after school closures ended. Interviewee 986 said “…there were two friends I used to hang about with a lot before the first lockdown but we never talked during the first lockdown and then we were back to school and I completely drifted away” (Female, 15 at T2, White, Mid to high SES, T2). This was further corroborated by interviewee 1504 who said “Friends contacted me by chat but as time went on we did not talk as much. I lost touch with some friends” (Female, 15 at T2, White, Mid to high SES, T2). For many adolescents, across all the groups, it was not possible to maintain all their pre-pandemic friendships during school closure and social isolation. The reduction in friendships was evident when schools reopened as they did not interact with such peers in the same manner. All adolescents reported that they lost connection with such pre-pandemic friends because of the reduced, or lack thereof, face-to-face interactions with peers.

However, adolescents in the ‘Enduring resilience’ and ‘Reaching resilience’ groups reported that the reduction in the quantity in friendships led to a re-evaluation and re-appraisal on what ‘friends’ or ‘friendships’ meant to them. A friend was someone who would support them in times of need or reached out to them throughout the pandemic. This was not observed in the ‘Declining resilience’ nor in the ‘Enduring non-resilience’ groups.

I think it was more because the more time we spent in lockdown, the more time you realised that oh she’s actually reaching out to me, she’s checking up on you and she’s actually there… while other people just like we left high school and there was just no contact at all (Interview 916, Female, 14 at T2, White, Mid to high SES, T2).

…I think I saw a lot of people who weren’t actually my friends. Because I think that one argument that I had with someone, I think it’s changed my whole perspective on it and I think if Covid wasn’t around, I wouldn’t have had that perspective because it would have never happened (Interview 817, Male, 17 at T2, White, Low SES, T2).

As a result, these adolescents became more appreciative of those who they considered to be their friends and, were active in developing better-quality and supportive relationship with peers who reciprocated. Interviewee said “…I could just be one phone call away and they would be there for me…” (Female, 14 at T2, White, Mid to high SES, T2). As a result, these young people were able to establish friendships that had stronger mutual connections and believed that they could rely on these peers during difficult times and were ready to do the same for them.

#### Quality of Family Relationships

The role of family relationships on adolescents’ mental well-being during different phases of the pandemic, especially during the various lockdowns, is complex. Firstly, for many adolescents (*n* = 22) family, especially parents, were supportive during the COVID-19 lockdowns. Interviewee 409 said “Overall, I feel I have coped well, with the support of family we have come out stronger both physically and mentally…” (Male, 17 at T2, White, Mid to high SES, T2).

However, amongst adolescents who reported good family relationships, there were significant group differences in how adolescents spoke of their experience with their family members during the pandemic. Compared to the other groups, adolescents in ‘Enduring Resilience’ and ‘Reaching Resilience’ groups described positive changes in their family dynamics which led to the development of supportive, open, and emotionally connective relationships across its members. Interviewee 409 said “Life during COVID was very different from what I had been used to before, although we had issues between siblings as a family, we have become closer and more appreciative of each other” (Male, 17 at T2, White, Mid to high SES, T2). This was further corroborated by interviewee 454 who said:

My relationship with my mum and dad is definitely very close much closer than before the pandemic as we were all at home a lot of the time together but instead of being sad my mum and dad encouraged us to do things like play board games… (Male, 15 at T2, White, Mid to high SES, T2).

Adolescents described that as a family there was an active drive to increase social engagement through planned and organic group activities. Family members of the adolescents were described as helpful during lockdown by temporarily reducing boredom through activities (walking or boardgames); being available to discuss problems; or promoting a sense of normality or structure. As a result, these adolescents voiced that, as a family, they felt closer and, that their conflict resolution skills and communication had improved. Interviewee 1539 said “…now we talk more, they try and sort things out without having arguments…” (Female, 14 at T2, White, Mid to high SES, T2).

In comparison adolescents in the ‘Declining resilience’ and ‘Enduring non-resilience’ groups reported that “nothing changed” (Interview 986, Female, 15 at T2, White, Mid to high SES, T2) when asked to describe their family dynamics during the COVID-19 pandemic. In one interview, the adolescent stated that their family members, whom they had good relations with, either kept to themselves or worked throughout the pandemic, and as a result, they felt an extreme sense of loneliness during all the lockdowns (Interview 1523, Female, 17 at T2, White, Mid to high SES). Therefore, for adolescents in the ‘Declining resilience’ and ‘Enduring non-resilience’ groups, their good pre-pandemic family relationships may not have been enough to manage the pandemic-related adversity.

#### Regaining a Sense of Control

Compared to the other groups, adolescents in the ‘Enduring resilience’ and ‘Reaching resilience’ groups reported how they were able to either regain or maintain a sense of control over their lives, including their mental well-being, during the pandemic.

I feel like I was mature, but I feel like now I’ve kind of seen like… because of the fact that I worked and I’ve seen how the actual big world works, I was like, okay, yes. It’s time to see what I can do and where I want my future to go (Interview 916, Female, 14 at T2, White, Mid to high SES, T2).

Pandemic-specific opportunities including having a job, learning a new hobby, or more broadly, having the time and freedom to self-reflect and self-discover. As a result, adolescents stated that such opportunities or freedom led to a greater sense of agency over their present situation, as well as their future. This includes the growing acknowledgement for the importance of looking after their own mental well-being. Interviewee 362 said “These days, I am doing better than I was before. I have learnt to focus and take care of myself better and to be less insecure of myself” (Female, 17 at T2, Asian/Asian British, Mid to high SES, T2). Therefore, in a time of uncertainty, young people in the ‘Enduring resilience’ and ‘Reaching resilience’ groups reported how they were able to regain a sense of internal control during the COVID-19 pandemic. This was not described by adolescents in the ‘Declining Resilience’ and ‘Enduring non-resilience’ groups.

### Examining Concordance between Quantitative and Qualitative Measures of Mental Well-Being and Resilience

For adolescents categorized (Table 1) as resilient (N at T1 = 15; N at T2 = 26) or non-resilient (N at T1 = 16; N at T2 = 5) for both the quantitative mental well-being (SWEMWBS) and resilience (CYRM) scores at T1 and T2 (see Table 2), t-tests revealed no significant difference between these groups (T1: SWEMWBS t(23) = 0.83, *p* = .41; CYRM t(23) = 0.04, *p* = .97, respectively; T2: SWEMWBS t(23) = 1.70, *p* = .10; CYRM t(23) = 0.20, *p* = .84, respectively).

Following this analysis, we then examined whether there was any congruency between the four resilience groups identified ((1) ‘Enduring resilience’. (2) ‘Reaching resilience’, (3) ‘Declining resilience’, and (4) ‘Enduring non-resilient’) and the quantitative self-report scores for well-being and resilience at T1 and T2 (Table 2). Again, contrary to expectations, a lack of congruency was found. Across all the four groups, mental well-being (as assessed by the SWEMWBS), and resilience (as assessed by the CYRM) did not significantly differ for adolescents during the initial (T1) and later phases (T2) of the COVID-19 pandemic. Thus, for the well-being scores (SWEMWBS), there were no significant differences between the two time points for all four groups (‘Enduring resilience’: t(10) = 1.32, *p* = .22; ‘Reaching resilience’: t(9) = 0.12, *p* = .90; ‘Declining resilience’: t(1) = 0.81, *p* = .57; ‘Enduring non-resilience’: t(1) = 0.35, *p* = .79). Similarly, for resilience (CYRM) scores, there were no significant differences between time points for all four groups (‘Enduring resilience’: (t(10) = 0.35, *p* = .74; ‘Reaching resilience’: t(9) = 0.66, *p* = .53; ‘Declining resilience’: t(1) = 0.60, *p* = .66; ‘Enduring non-resilience’: t(1) = 1.00, *p* = .50).

## Discussion

The current investigation provides valuable insights into different trajectories of resilience; measures of resilience; and factors that encourage resilience in adolescents over a two-year period during the COVID-19 pandemic. A key finding is that the majority of adolescents who took part in this study either had enduring (42%) or reached resilience (42%) during the COVID-19 pandemic. This is not consistent with existing literature which suggests a growing concern for lasting poor mental well-being and resilience due to the adverse impact of the COVID-19 pandemic amongst adolescents (Dewa et al., 2021; Imran et al., 2020; Lee, 2020; Orben et al., 2020; Widnall et al., 2022). Instead, as demonstrated in adults (Shevlin et al., 2023a, b), our findings show that many adolescents were able to use available internal or external resources to manage, counteract or outweigh the challenges presented by the COVID-19 pandemic. At the same time however, there were a small number of adolescents who endured non-resilience (three out of thirty-one) or experienced declining resilience (two out of thirty-one) across the COVID-19 pandemic. This small group of adolescents were experienced poor mental well-being as a lasting impact of the COVID-19 pandemic (two years after the initial wave), and hence it is crucial to identify these adolescents from the general population for further monitoring of their mental well-being post pandemic; as well as, to provide support and interventions to combat the impact of the COVID-19 pandemic.

Another key aim of the current investigation was to identify factors that encourage resilience during the COVID-19 pandemic and understand how, or if, they changed across the trajectories of resilience. Firstly, as supported by previous literature (Larivière-Bastien et al., 2022; Widnall et al., 2022), quality, rather than quantity, of friendships may promote positive mental well-being in adolescents during the COVID-19 pandemic. The current study builds upon this further by demonstrating that not all adolescents were able to establish high quality friendships during the COVID-19 pandemic and consequently, such adolescents were not able to endure or reach resilience. Hence, quality of friendships is not only likely to promote good mental well-being but may play an important role in encouraging resilience in adolescents despite the reported difficulties in maintaining friendships during lockdown, social isolation, and school closures.

Secondly, quality of family relationships may have contributed to adolescents’ resilience across the COVID-19 pandemic. In those who showed either enduring or were reaching resilience, these adolescents reported that the family dynamics changed and adapted as a response to the pandemic-related adversity and as a result, became an effective resource for promoting good mental well-being. In the declining resilience and enduring non-resilience group, however, adolescents reported good pre- and post-pandemic family relationships but also stated that the family dynamics did not change during the COVID-19 pandemic. Therefore, it may be that the family’s adaption to manage the pandemic-related adversity is crucial for adolescents’ resilience, rather than good pre-pandemic family relationships. Outside the context of the COVID-19 pandemic, this process has been coined as ‘family resiliency’ (McAuley et al., 2012), where family dynamics adapt to respond to, and protect its members from adversity. Further research is needed to understand how family resiliency occurs, or can be encouraged, under the context of lockdown and social isolation.

From a developmental perspective, the importance of family relationships was unsurprising, especially for the age range of those interviewed in the current investigation (T1: aged 13–16; T2: aged 14 and 17), as they are still living within the family unit. During adolescence, there is a natural inclination among individuals to pursue independence from their families (Albarello et al., 2018; Avedissian & Alayan, 2021; Hay & Ashman, 2003; Roach, 2018; Spear & Kulbok, 2004; Steinberg & Morris, 2001). This drive stems from the desire to cultivate autonomy and competency, which not only contributes to their well-being during this developmental stage but also sets the foundation for later stages of development (Avedissian & Alayan, 2021; Hay & Ashman, 2003; Roach, 2018). That, however, does not imply that family becomes unimportant at this life stage; quite the contrary. In fact, family remains a crucial source of support and guidance for adolescents (Barrera Jr & Li, 1996; Triyanto & Iskandar, 2014; van Harmelen et al., 2016; Wills et al., 2014). Moreover, various dependencies, including financial stability, access to nutrition and housing, and the presence of emotional warmth, are inherent during the adolescent period. (Morris et al., 2017; Patalay & Fitzsimons, 2018; Szwedo et al., 2017). Therefore, similar to the pre-pandemic literature, the current study demonstrates that healthy family functioning during adolescence likely predicts good wellbeing under the context of the COVID-19. For now, the findings from the current study argue that for future pandemics there should be a focus on encouraging supportive connections between adolescents and their families; to promote a healthy dependency and aid the process of resilience in such adolescents.

Thirdly, this investigation is the first to our knowledge to identify the importance of regaining a sense of control for adolescents’ resilience during the COVID-19 pandemic. Adolescents who endured or reached resilience reported that the pandemic led to new opportunities or the freedom to work; re-evaluate what was important to them; or gained new skills and hobbies. This allowed them to regain a sense of control, or agency, during uncertain times. This regaining control in an uncertain context was reported to be important for adolescents who were able to endure or reach resilience during the pandemic. However, more research is needed to clarify the link between regaining control and adolescents’ resilience during the COVID-19 pandemic. Currently, it is uncertain if adolescents were reporting their re-appraisal and new meaning of their pandemic experience as an effect of their resiliency; or, if they developed internal resources, such as greater internal locus of control or coping mechanisms to manage the COVID-19 adversity.

The final aim of the current investigation was to examine the concordance between quantitative and qualitative measures of mental well-being and resilience. Findings from the current investigation revealed that self-reports (well-being as measured by SWEMWBS and resilience as measured by the CYRM) could not distinguish between adolescents who were identified as being resilient or not at either time point (T1 or T2) based on their qualitative data. Additionally, for adolescents who voiced a change qualitatively between T1 and T2 (‘Reaching resilience’ and ‘Declining resilience) self-reports (SWEMWBS and the CYRM) also did not reflect this change. Instead, all groups reported high scores, indicating good mental well-being and resiliency as measured by self-report questionnaires. This suggests that identification of vulnerable adolescents during the COVID-19 may not be possible through self-reports alone, despite self-report questionnaires having been the prevailing method for studying the impact of COVID-19 on adolescents and young people globally. The latter suggests that caution is needed when drawing conclusions from self-reports alone as these may not always accurately reflect adolescents’ COVID-19 experience.

The lack of congruency between our qualitative and quantitative data may be because they tap into different constructs or experiences, and this lends support for the need to employ both longitudinal qualitative and quantitative methods to assess both adaptive and maladaptive impacts of adversity on mental well-being and resilience on adolescents. It is possible that self-report questionnaires do not accurately capture the more nuanced complexity or subtlety of a person’s psychological state (Deighton et al., 2014). Further, our qualitative measures relied on adolescents’ retrospective accounts to understand their mental well-being during the initial months of the COVID-19 pandemic (T1), which could be prone to inaccuracies due to recall bias (Coughlin, 1990).

Regardless, our concern is that researchers and UK policies often rely on self-report data alone to inform knowledge and policy regarding the psychosocial impact on adolescents during the COVID-19 pandemic (Creswell et al., 2021; Kauhanen et al., 2022; Office for Health Improvement and Disparities, 2022). Future research is needed to determine why, and to what extent there is a mismatch between qualitative and quantitative self-reports on adolescents’ COVID-19 experience. Additionally, there is a need to know if the findings extend beyond the self-reports used in the current investigation: SWEMWBS and the CYRM. For now, caution is advised when interpreting adolescents’ COVID-19 experience from self-report questionnaires alone as doing so may obfuscate their true experiences.

### Strengths and Limitations

The major strength of the current investigation is the mixed-methods longitudinal design of the study. Firstly, the longitudinal design allowed insights into the different trajectories of resilience in adolescents over a two-year period. Moreso, the longitudinal qualitative interviews enabled young people to narrate their own COVID-19 story and provide insightful reflections on their journey. This is a key strength as the COVID-19 pandemic was dynamic in nature, and how adolescents adapted to this ever-changing global event is not yet fully understood. Therefore, the longitudinal approach enabled us to identify trajectories of resilience and understand which factors played a role in adolescents’ resilience from the initial months of the pandemic (April 2020) to two-years following (January to May 2022).

Secondly, the current investigation adopted a mixed-methods approach to provide a deeper and fuller understanding of adolescents’ COVID-19 experience. The interviews allowed adolescents the opportunity to voice their experience; and to report what was important for their mental well-being and how this changed across the pandemic. At the same time, the self-reported data, using standardised measures, enabled us to explore the degree to which these might reflect mental well-being and resilience in adolescents in the context of the COVID-19 pandemic. Therefore, the mixed-methods approach has enhanced our knowledge of adolescents’ resilience between the initial months of the COVID-19 pandemic to two-years following.

However, this study is not without its limitations. Due to the nature of the mixed-methods approach, small sample sizes for the quantitative analysis could have obscured significant changes in well-being and resilience scores. This means that there is a risk of not detecting a significant result, and as such, a true effect. Additionally, due small sizes the power to perform such statistical tests is questionable. Therefore, caution is needed when interpreting and generalising the quantitative results. There is also a need for future research to attempt to replicate the findings using larger sample sizes. By contrast, the sample was large for qualitative analysis. Yet, caution is also advised when generalising the qualitative findings to all adolescents living the UK. Despite best efforts during the recruitment of this study by prioritising recruitment of adolescents from ethnic diverse and low SES backgrounds, the qualitative sample may still be biased towards adolescents who are White British and, or from middle to high SES backgrounds. Furthermore, unlike the Enduring and Reaching resilience groups (*n* = 13 for both groups), data saturation was not achieved for adolescents who were selected as either ‘Declining resilience’ (*n* = 2) or ‘Enduring non-resilience’ (*n* = 3) due to the nature of the recruitment strategy. Despite best efforts many adolescents declined the opportunity to be interviewed (over 150) and recruitment ended once all eligible adolescents were invited to participate (*n* = 212) rather than reaching saturation.

Overall, there is need to continue to understand the process of resilience in adolescents during pandemic-related adversity. Although the current investigation provides valuable insights, there is scope for much more research in this area so that practical implications can be drawn. Future investigations could focus on identifying strategies to reduce technological barriers in peer interaction during school closures; as well as draw upon multiple family members in their investigation, rather than a specific focus on a single member to understand family dynamics during the pandemic.

## Conclusion

Our research provides an in-depth overview of the trajectories of resilience in adolescents living in the UK during the COVID-19 pandemic. A key finding is that most adolescents we interviewed showed enduring or reached resilience during different phases of the COVID-19 pandemic. Simultaneously, and significantly, there were a small number of adolescents who reported lasting poor mental well-being due to the pandemic, and this sub-group should be the focus of support efforts. In addition, the current investigation provides valuable insight into the factors that encouraged resilience in adolescents during the COVID-19 pandemic: quality of friendships, quality of family relationships, and regaining a sense of control. In the event of future pandemics, it may be crucial to devise strategies to encourage the likelihood of such factors so that more adolescents showed enduing and/or were able to reach resilience under conditions of adversity. Lastly, whilst there is a key need to identify adolescents who are not resilient, from those who were, and have suffered long-lasting adverse outcomes due to the pandemic, quantitative findings suggest that self-reports alone may not be able to do so.

## Electronic Supplementary Material

Below is the link to the electronic supplementary material.

Supplementary Material 1

Supplementary Material 2

## Data Availability

The data collected in this investigation has been made freely available for researchers and non-commercial use through the UK Data Service (identifying code: SN 9018).
