# Supplementary material for: “It’s Time to see What I Can Do”: A Mixed-Methods Investigation into Trajectories of Resilience in Adolescents during the COVID-19 Pandemic
Source: J Child Adolesc Trauma. 2024 Jun 5;17(4):1091–103. doi: 10.1007/s40653-024-00642-5 (PMC11646241; doi:10.1007/s40653-024-00642-5)
Supplement: Supplementary file 1 — Supplementary Material 1 [file 40653_2024_642_MOESM1_ESM.docx]

**Supplementary material**

**Verbal Interview Guide**

**Section A (life before COVID-19)**

***General***

Tell me about what was going on in your life before COVID-19.

How old were you then? What school year were you in?

What kinds of things were you doing?

On an average day what was your routine like?

What was going on in your life then?

What did you enjoy doing?

***School***

Did you take part in any extracurricular activities like sports or after-school clubs?

Home life/outside school

Who were you living with?

Parents/caregivers - do they work? What do they do?

Siblings - how old?

What did you like to do in your free time?

What did you like to do with your friends?

***Mood***

How did you feel around that time?

Were you happy/sad?

Do you remember if you were having any problems before the pandemic, or if there is anything you were worried about at that time?

**Section B (first lockdown in March/April 2020)**

***First hearing about COVID***

Can you remember when you first heard about COVID-19?

Is there anything that stands out in your mind as a distinctive moment around then?

[If indicating that they first thought it would be short-lived] Can you remember when you first realised it was a bit more serious than you first thought?

How did you feel about it?

Were you worried about catching COVID?

Were you worried about people you know catching COVID?

***First lockdown***

Could you tell me about your thoughts and feelings when you learned about the first lockdown in March 2020?

What can you remember about the first lockdown?

What would a typical day have been like during the first lockdown?

[If indicating they were worried/stressed] Can you remember how you tried to manage or cope with the situation or how you were feeling?

How did you feel about being away from your friends?

How did you feel being with family?

***School/routine during the first lockdown***

What was studying at home or at school like?

Did you try to create a routine or timetable to stick to, or did you just sort of take each day as it came?

What was your motivation like for schoolwork?

Would you have liked more/less structure?

[If child of key worker] How did you feel about still going into school when some people were studying at home?

Looking back now, what do you think was the worst thing about being in lockdown?

If we contrast that, were there any good things about being in lockdown?

Eat Out to Help Out scheme

So after that first initial lockdown, some restrictions were lifted, and over the summer we had things like the Eat Out to Help Out scheme and some sort of return to normality, but there were still lots of restrictions and measures implemented by the government to try and keep us safe, so things like social distancing and wearing masks indoors. How did you feel about this?

***Other lockdowns***

As we moved into autumn 2020, COVID cases began to rise again and we had the tier system put in where some places had more restrictions than other places and then we eventually went into another national lockdown in November 2020, and then things kind of lifted over Christmas but not really and then we want back into another lockdown in January. Can you remember how you felt about these lockdowns?

If at all, how did they feel different to the first lockdown?

**Section C (present situation - returning to ‘normal’)**

***General***

How would you say things are now?

What were you most excited about being able to do again?

What, if anything, were you worried about in returning to the new normal?

Does COVID still have an impact on your day-to-day life?

Is COVID still something you think about?

***School***

How did you feel about returning to school/college/work?

Did you feel it was safe to go back?

[If they didn’t think it was safe] How did you manage that conflict between not thinking it was safe but still having to go in?

***Vaccine***

How did you feel when you heard you were eligible to get the vaccine?

[If they’ve had the vaccine] Do you feel different now that you've had the vaccine?

**Section D (reflection and resilience)**

***Coping***

How do you feel you coped with the pandemic?

Do you think you coped well, or did you find it quite difficult?

Why do you think this?

Is there anything that could have made it easier/better for you?

Tell be about your sleeping routine across the pandemic?

***Relationships***

Who was most helpful to you during this time? And why?

Do you think your relationships with your friends have changed because of COVID-19?

Do you think your relationships with your family have changed because of COVID-19?

***Government***

How did you feel about how the government handled the situation?

Is there anything you think the government could have done differently that would have made life easier/better for people your age during the pandemic?

***General***

Do you feel different as a person now compared to before the pandemic?

Is there anything that you miss about pre-pandemic life?

Do you feel you have missed out on somethings because of the pandemic? If so, what, and how?

Are there any important lessons that you learned through experiencing the pandemic?

Were there any good things to come out of the pandemic?

I hope we don’t, but if we were to have another pandemic in 50 years, and we went into a nationwide lockdown again, what advice would you give to someone the same age as you are now, who are just about to experience their first lockdown?

Looking back now, what do you think was the worst or most difficult thing about the past 18 months of the COVID pandemic?

***Closing Questions***

So what are your hopes or aspirations for the future?

Has this changed from your hopes pre-COVID or is it still the same?

Have you got any worries about the future?

Has anything been brought to your mind that maybe you hadn’t thought of before this interview?

Is there something else you think I should know to better understand your experiences of the COVID-19 pandemic?

Is there anything else you would like to ask me before we finish?

**Written Interview Questions**

Tell me about what was going on in your life before COVID-19.

Tell me about what school was like before COVID-19.
​​​​​ *For example, tell us what you enjoy or dislike about school or if you took part in any extracurricular* activities*like sports or after-school clubs*

Tell me about your friends/friendships before COVID-19.
 *For example, tell us if you had small or large friendship group, or, if you did, what you and your friends did outside of school.*

Tell me about your home life before COVID-19.
 *For example, tell us about who you lived with and what your relationship with them is like or tell us about any activities you like to do at home.*

Tell me about your mood before COVID-19.
​​​​​​ *For example, tell us if you were generally happy or sad, or tell us about any problems or worries or exciting plans you had before the pandemic.*

Please use this space to add anything else that would help us to understand your life before the COVID-19 pandemic.

*If you don't have anything further to add just click here to move to the next section*

Tell us about when you first heard about the COVID-19 virus. 
This time period focuses on before the first UK national lockdown, which started in mid-March 2020

*For example, tell us how you or others felt when you first heard about the virus or if you were worried about the virus (either for yourself or others) or tell us about how, if it did, social media/the news made you feel about the virus.*

Tell us about what it was like for you during the first UK national lockdown - From Mid-March 2020 to June 2020 - Seems ages ago now! .

*For example, tell us about your thoughts and feelings during this time, or tell us how your routine changed, or tell us about the good and bad bits about the first lockdown.*

Tell us about what your school life was like during the first lockdown.

*For example, tell us what studying at home like (or studying at school whilst everyone else was at home!), or tell us about the good and bad bits of studying at home, or tell us what could have made it better or easier for you.*

Tell us about what your friendships during the first lockdown.

*For example, tell us about what it was like not being able to see your friends or tell us, if you did, about how you kept in contact with your friends, or tell us, if it did, how your friendships changed during this time.*

Tell us about your home life during the first lockdown.

*For example, tell us what is was like being with your family/household, or tell us about any changes to your household (parents, siblings, grandparents, pets) during this time.*

After the first UK lockdown, some restrictions were lifted over the summer and we sort of returned to normal. However, there were still a lot of restrictions and measures still implemented to keep everyone safe (face masks and social distancing).
How did you feel about this?

*For example, tell us about any activities you did during this time that you enjoyed or did not enjoy, or tell us about how you felt about the measures that were implemented at this time or tell us, if it did, how it felt different compared to being in lockdown.*

As we move into autumn 2020, COVID-19 cases began to rise again and we had the tier system (where some places had more restrictions than other places), and then we went into another lockdown in November 2020, then we had restrictions over Christmas, followed by another lockdown in January.
Can you remember how you felt during these lockdowns?

*For example, tell us, if it did, how the lockdowns felt differently to the first lockdown and why, or tell us if anything changed (home life, school-life, friendships your mood/feelings) during all the other lockdowns.*

Please use this space to add anything else that would help us to understand your life during the COVID-19 pandemic.

*If you don't have anything further to add just click here to move to the next section*

Tell about your thoughts and feelings when the restrictions began to lift and we began to return to 'normal'.

*For example, tell us about if you were worried or excited about anything, or tell us if you felt safe or unsafe about anything when returning to the new normal.*

Tell me about your life now.

*For example, tell us about your routine now, or tell us about your general mood (happy/sad/anxious/excited) now, or tell us about anything that is different now because of the pandemic, or tell us about, if it does, how COVID-19 still impacts your everyday life.*

Tell me about your school life now.

*For example, tell us about your school work or motivation for your school work is like now compared to during or before the pandemic, or tell us about your teachers (are they supportive/strict/understanding/being unfair), or tell us, if you did, what the transition from school to sixth form/college was like.*

Tell me about what your relationships are like now.

*For example, what is your relationship with your friends/parents/siblings/grandparents like now.*

How did you feel when you heard you were eligible to get the vaccine?

*For example, tell us how you feel about yourself or others (friends/family) getting or having the vaccine, or tell us, if you did hear any, how did the rumours about the vaccine made you feel.*

Please use this space to add anything else that would help us to understand your life now.

*If you don't have anything further to add just click here to move to the next section*

Tell us about your thoughts and feelings about the pandemic overall?

*For example, tell us about the best or worst things about or to have come out of the pandemic for you, or tell us if you felt you missed out on or learnt something because of the pandemic, or tell us if you feel different as a person now compared because of pandemic.*

How do you feel you have coped with the pandemic?
​​​​​​​ *For example, tell us about why you feel you coped or did not cope well with the pandemic, or tell us about what did or could have helped you to cope better, or tell us if catching the virus yourself, or someone close to you, changed how you coped with the pandemic.*

How do you feel about how the UK Government has handled the pandemic?

​​​​​​​*For example, tell us if you feel they handled it well or badly and why, or tell us how you feel about the scandals that have come out of the pandemic (such as the Christmas/lockdown parties), or tell us about how the constant changes in information and, or rules made you feel, or tell us if you think the Government could have done to have made life easier/better for you, during the pandemic.*

Who was most helpful during the pandemic, and why?

If we were to have another pandemic in 50 years or so, and we went into a national lockdown again, what advice would you give to someone the same age as you were, who are about to experience their first lockdown?

Thinking about the future, what are your hopes for the future?
​​​​​​​ *For example, tell us about your hopes for your future, or tell us if your hope for the future has changed because of the pandemic, or tell us about anything you are worried or excited about in the future.*

Is there anything else you think we should know to better understand your experience of the COVID-19 pandemic?

If you don't have anything further to add just click here to move to the next section

**Resilience category for each interview**

**Table 1.**

*Resilience categorisation for T1 and T2 for each interview, including demographics.*

| ID | Age | Gender | Ethnicity | SES index | Interview | T1 Category | T2 Category |
| --- | --- | --- | --- | --- | --- | --- | --- |
| 189 | 17 | Female | White | Mid/high SES | Written | Non-resilient | Resilient |
| 362 | 17 | Female | Asian/Asian British | Mid/high SES | Written | Non-resilient | Resilient |
| 367 | 16 | Female | Asian/Asian British | Mid/high SES | Verbal | Non-resilient | Resilient |
| 409 | 17 | Male | White | Mid/high SES | Written | Non-resilient | Resilient |
| 410 | 15 | Male | White | Mid/high SES | Written | Resilient | Resilient |
| 412 | 16 | Male | White | Mid/high SES | Written | Non-resilient | Non-resilient |
| 454 | 15 | Male | White | Mid/high SES | Written | Non-resilient | Resilience |
| 569 | 16 | Female | White | Mid/high SES | Written | Non-resilient | Resilient |
| 779 | 16 | Male | White | Low SES | Written | Non-resilient | Non-resilient |
| 781 | 14 | Male | White | Low SES | Written | Resilient | Non-resilient |
| 817 | 17 | Male | White | Low SES | Written | Resilient | Resilient |
| 911 | 17 | Female | White | Mid/high SES | Written | Non-resilient | Resilient |
| 916 | 14 | Female | White | Mid/high SES | Verbal | Resilient | Resilient |
| 986 | 15 | Female | White | Mid/high SES | Verbal | Non-resilient | Non-resilient |
| 993 | 15 | Female | White | Mid/high SES | Written | Non-resilient | Resilient |
| 1126 | 14 | Male | Black / African / Caribbean / Black British | Low SES | Verbal | Resilient | Resilient |
| 1261 | 16 | Female | Black / African / Caribbean / Black British | Low SES | Verbal | Non-resilient | Resilient |
| 1329 | 15 | Female | White | Mid/high SES | Written | Resilient | Resilient |
| 1349 | 17 | Female | White | Low SES | Written | Resilient | Resilient |
| 1353 | 17 | Female | White | Mid/high SES | Written | Non-resilient | Resilient |
| 1407 | 15 | Female | White | Mid/high SES | Verbal | Resilient | Resilient |
| 1501 | 15 | Female | White | Mid/high SES | Written | Resilient | Resilient |
| 1504 | 15 | Female | White | Mid/high SES | Written | Resilient | Resilient |
| 1523 | 17 | Female | White | Mid/high SES | Verbal | Non-resilient | Resilient |
| 1539 | 14 | Female | White | Mid/high SES | Verbal | Resilient | Resilient |
| 1584 | 15 | Female | White | Mid/high SES | Verbal | Resilient | Non-resilient |
| 1714 | 15 | Male | Black / African / Caribbean / Black British | Low SES | Verbal | Resilient | Resilient |
| 1765 | 15 | Male | White | Mid/high SES | Written | Non-resilient | Resilient |
| 1788 | 14 | Male | White | Low SES | Written | Resilient | Resilient |
| 1790 | 17 | Male | White | Mid/high SES | Written | Non-resilient | Resilient |
| 1929 | 17 | Male | White | Mid/high SES | Written | Resilient | Resilient |

*Note:* SES = Socioeconomic Status
